# Partnership for the implementation of mental health policy in Nigeria: a case study of the Comprehensive Community Mental Health Programme in Benue State

**DOI:** 10.1186/s13033-020-00344-z

**Published:** 2020-02-21

**Authors:** G. K. Ryan, E. Nwefoh, C. Aguocha, P. O. Ode, S. O. Okpoju, P. Ocheche, A. Woyengikuro, J. Abdulmalik, J. Eaton

**Affiliations:** 1 Faculty of Epidemiology and Population Health, London School of Hygiene and Tropical Medicine, Keppel Street, London, WC1E 7HT UK; 2 Centre for Global Mental Health, London School of Hygiene and Tropical Medicine, Keppel Street, London, WC1E 7HT UK; 3 CBM International, Stubenwald-Allee 5, 64625 Bensheim, Germany; 4 Department of Medicine, Imo State University, Okigwe Road, Ugwu Orji, Owerri, Nigeria; 5 Benue State Comprehensive Community Mental Health Programme, Depot Road PMB 0360, Otukpo, Nigeria; 6 Department of Psychiatry, College of Medicine, University of Ibadan, University College Hospital, PMB 5116, Oyo, Nigeria

**Keywords:** Global mental health, Community mental health, Primary care, Scale-up, Case study, Nigeria

## Abstract

**Background:**

71% of countries in the World Health Organisation’s (WHO’s) African Region have a stand-alone mental health policy or plan, but only 14% have fully implemented it. In Nigeria, integration of mental health into primary care has been a stumbling block to the implementation of the 1991 National Mental Health Policy, 2013 Policy on Mental Health Services Delivery and the National Mental, Neurological and Substance Use Programme and Action Plan. A partnership between public and private not-for-profits in Benue State, the Comprehensive Community Mental Health Programme (CCMHP) has successfully integrated mental health into primary care in alignment with the national mental health policy and the WHO’s mental health Gap Action Programme Intervention Guide (mhGAP-IG). There is a need to document such examples in order to inform policy implementation in Nigeria and other low- and middle-income countries (LMICs).

**Methods:**

We followed the Case Study Methodology to Monitor and Evaluate Community Mental Health Programmes in LMICs. Four field visits were conducted between 2013 and 2017 to document the first phase of activities of CCMHP, covering the period of January 2011 through June 2016.

**Results:**

In its first phase, CCMHP trained 19 community psychiatric nurses and 48 community health extension workers in mhGAP-IG, establishing 45 new mental health clinics in primary care facilities across Benue, a state more populous than many countries. As a result, 13,785 clients (55% male, 45% female) were enrolled in mental health services either in primary care or in one of two pre-existing community-based rehabilitation facilities. Most are adults over age 18 (82.75%), and present to services with epilepsy (52.38%) or psychosis (38.41%).

**Conclusion:**

The case of CCMHP demonstrates it is possible to rapidly scale-up mental health services in line with national mental health policy using the mhGAP-IG, even in a challenging, low-resource setting. Multi-sectoral partnerships may help to overcome some of the barriers to successful integration of mental health into general healthcare by capitalising on the resources and expertise of both state and non-state actors. However, a difficult political context could threaten the sustainability of the programme if funder requirements force a rapid transition to full government ownership.

## Background

### Mental health policy and implementation in Nigeria

The 1991 National Mental Health Policy for Nigeria declared that mental health should be integrated into general health services at all levels [1]. The policy placed much of the responsibility for ensuring comprehensive access to mental health care on primary care, and thus on local governments [2–4]. The 2013 Policy on Mental Health Services Delivery and the National Mental, Neurological, and Substance Use Programme and Action Plan for Nigeria both reaffirm this commitment to the provision of mental health services in primary care [5]. However, these policies have never been fully implemented [2, 6, 7]. Indeed, the World Health Organisation’s (WHO’s) 2014 Atlas indicated that while 71% of countries in the African Region had a stand-alone mental health policy or plan, only 14% had fully implemented it [8].

Saraceno and colleagues (2007) attribute what they call the “ill fated” integration of mental health into Nigeria’s primary care system to three overarching issues: inadequate training and supervision of primary care workers, insufficient funding and lack of political will [7]. A more recent study conducted in the southwest of Nigeria supports these observations and also points to knowledge and attitudes about mental health as well as unreliable supply chains [2]. These issues are not unique to Nigeria, or to mental health specifically [7, 9, 10]. For example, insufficient and inequitable distribution of the workforce and lack of essential medicines have both been cited as barriers to the effective implementation of primary care more generally in Nigeria [11].

The private, non-state sector, which includes commercial interests as well as not-for-profits such as faith-based, community-based and non-governmental organisations (NGOs), fills some of the gaps in Nigeria’s primary care system [12]. Consequently, the 2013 Policy on Mental Health Services Delivery encourages “Public Private Partnership (including NGOs)… as a working model for providing, and funding, services” (pp. 22) [5].

### Public-private partnerships in health care delivery

A public–private partnership (PPP) is “a long-term contract between a private party and a government agency, for providing a public asset or service, in which the private party bears significant risk and management responsibility” [13, 14]. While some authors limit their definition of PPPs to those involving for-profit entities [15, 16], others include contracts between public agencies and private not-for-profits [17, 18].

The origin of PPPs is often traced back to the 1970s and '80s, when multiple recessions and growing public debt led governments to pursue more private-sector investment in infrastructure development and service delivery [19]. The United Kingdom (UK) was one of PPPs’ early adopters, launching its Public Finance Initiative in 1992 [20]. By 2001, nearly 450 PPP contracts had been signed, at a value of more than 20 GBP billion [21]. By 2006, the value of health contracts alone reached 3 GBP billion [22]. In general, low-income countries have been slower to adopt PPPs than some middle-income countries (notably China, India and Russia) and many high-income countries (particularly Australia, United States, Portugal and other European countries). However, over the past decade governments have increasingly been turning to PPPs to circumvent budgetary constraints in low-resource settings [19].

In LMICs, PPPs for health take many different shapes [13, 23]. For example, a World Bank briefing for the 2013 Africa Health Forum divides PPPs into five categories: public health services (an apparent catch-all for partnerships with the private sector for provision of clinical and/or nonclinical public services); co-location (private wing or department in a public hospital); hospital services (private management of a public hospital); facilities-finance (private financing, construction and ownership of a hospital that is leased back to government); and combined facilities and services (a combination of the latter two categories) [18]. Meanwhile, much of the literature on PPPs in global health focuses on large international consortia such as the Global Alliance for Vaccines and Immunisations (GAVI) and the Global Fund to fight AIDS, TB and Malaria (“The Global Fund”) [15, 23–25]. These global health partnerships are sometimes categorised by the locus of management: a secretariat within an intergovernmental agency like the WHO; a not-for-profit host like the Task Force for Global Health; or a separate legal entity like the International AIDS Vaccine Initiative [23, 25]. However, significant ambiguity remains, despite efforts to more clearly define and characterise PPPs in the health literature.

Reflecting this general ambiguity on PPPs, Nigeria’s 2013 policy outlines a number of different contributions that NGOs, religious organisations and other not-for-profits can make to implementation and governance, but provides no guidance on—or examples of—how to structure PPPs in order to actually facilitate these contributions [5]. As Nakimuli-Mpungu and colleagues (2013, n.p.) note in their evaluation of a PPP in Uganda, “Unfortunately, PPPs to strengthen mental health services have not been well described in LMICs” [17]. There are surprisingly few documented examples from which to draw lessons for mental health policy in low-resource settings. In fact, much of what is known comes from high-income countries; the United States and United Kingdom account for nearly two-thirds of all publications on PPPs in health [13].

### Purpose of this case study

Our aim is to help inform the utilisation of PPPs for mental health policy implementation in Nigeria and other low-resource settings by documenting a promising example from Benue, a largely rural state in the North Central region with a population larger than many countries. We present a case study of the development and first phase of implementation (2011–2016) of the Benue State Comprehensive Community Mental Health Programme (CCMHP). Over this five-year period, the programme demonstrated that it was feasible to rapidly scale up mental health services in primary care through a PPP capitalising on the resources and expertise available within and outside of Nigeria’s public sector. Our case study describes what was achieved and how, identifies strengths and weaknesses observed in CCMHP’s first phase, and considers opportunities and threats that the programme can expect to encounter in future phases of implementation.

## Methods

Case studies are among the most common approaches to data collection on PPPs in health [13]. However, Roehrich, Lewis and George (2014) note that the methodology is often unstandardised and poorly described in this literature [13]. We therefore chose to follow a manualised case study methodology developed by the Case Studies Project of the Centre for Global Mental Health at London School of Hygiene and Tropical Medicine (LSHTM) and the disability and development organisation CBM [26]. This methodology is designed to organise and integrate information from a variety of different primary and secondary data sources across 14 domains of interest arranged in a tabular format (Table 1). Where possible, data collection should include participant observation carried out over a series of field visits, during which informal conversations, observations and personal reactions can be captured through field notes. In the process of data collection, the investigator is instructed to keep in mind the overarching research questions: “Is this programme working? Why or why not?” The investigator then draws on these insights and the information gathered in the domain table to develop a narrative description of the programme and a SWOT (strengths, weaknesses, opportunities and threats) analysis. This methodology has been used previously to document community mental health programmes in Sub-Saharan Africa, South and Southeast Asia [26–28].

**Table 1:** Overview of LSHTM-CBM case study methodology’s domains (Adapted from Cohen et al. [28])

Domain type	Domain
Context	Environment in which the programme functions
	Health system in which the programme functions
History	History of the programme
Programme model	Programme conceptual framework
	Engagement with broader systems
Programme organisation	Programme resources
	Programme management
Client populations	Client characteristics
	Pathways to care
Interventions	Clinical interventions
	Medications
	Psychosocial interventions (including self-help groups and livelihood programmes
	Accessibility of services
Information	Information system

### Qualitative

An external investigator (GKR) completed the domain table over the course of four field visits between 2013 and 2017. Each visit spanned two to six weeks. Semi-structured interviews were employed during the first visit and recorded via hand-written notes. Participants included the Project Coordinator, Community Mental Health Project Officer, Self-Help Group Development Project Officer, and six community psychiatric nurses (CPNs): two from community-based rehabilitation centres and four trained by the programme to serve in new clinics. All four field visits involved a combination of desk review and observation of programme activities captured through photographs (with written consent from identifiable human subjects) and field notes. Activities included: programme staff and stakeholder meetings; routine supervision visits to community-based rehabilitation centres, outpatient clinics and self-help groups; and trainings on leadership and advocacy for self-help groups and on monitoring and evaluation (M&E) for community psychiatric nurses, M&E officers and local government officials. Stakeholders involved in meetings included: the CBM Mental Health Advisor for Nigeria; welfare officers from community-based rehabilitation centres; the Bishop of the Methodist Church Diocese of Otukpo in Benue State; the Benue State Health Management Information Systems Officer; the Benue State Director of Public Health; and other state and local government officials. Where necessary, additional information was gathered via literature searches, document review, email and Skype discussions with programme staff.

Qualitative data collected on the first field visit were transcribed from hand-written notes and other documents and uploaded to Dedoose, an online qualitative research software developed by Socio Cultural Research Consultants, LLC. Data were coded deductively, using the domain table as the basis of a coding framework, by the lead author. Narrative summaries of the data were then entered into the relevant cells of the domain table. Data collected on subsequent visits were hand-coded and added to the table.

### Quantitative

Data on service utilisation were routinely collected by frontline providers and reported to CCMHP on a monthly basis using the programme’s MIND ME (Mental health INformation anD Monitoring and Evaluation) system, which several authors helped to develop and put in place (GKR, EN, POO, PO, SOO, AW, JE) in collaboration with other staff of CCMHP and the Benue State Ministry of Health and Human Services [29, 30]. CCMHP programme staff entered data from all 47 mental health clinics’ monthly reports for the period from January 2011–December 2016 into Excel spreadsheets for data cleaning and analysis. Clinical and demographic characteristics of 13,785 clients were analysed by summarising descriptive statistics for sex, age, and diagnosis, where available. Number of referrals in and out of each clinic were also calculated.

### Limitations

As described by the authors of the Case Study Methodology, its strengths lie in its ability to probe “phenomena as they occur or exist in real-life contexts” [26]. However, this case study also has several important limitations. First, although CCMHP has processes in place for quality assurance, data quality has not been formally assessed, and there is evidence of missing data (Table 2). Second, although CCMHP collects routine outcome data from clients, the programme does not currently have sufficient human resource capacity to process this data for analysis. As a result, it is not possible at this stage to carry out a before-and-after evaluation. Third, the position of the authors, many of whom work for or in close collaboration with CCMHP, could introduce bias. Fourth, we did not have the resources to audio-record and transcribe interviews, and note-taking is not the most reliable way to capture qualitative data. Fifth, due to language barriers, interviews were not conducted with programme beneficiaries. The outcomes and perspectives of CCMHP beneficiaries should be the subject of a future research evaluation using more rigorous methods.

**Table 2:** Implementation of Nigeria’s Mental Health Policy on Primary Care in Benue State (adapted from FMOH 2013)

Policy directive	CCMHP approach
1. Supervision A psychiatric nurse should ideally be posted to each health centre and supervised by a doctor in primary care, who is in turn supervised by a community psychiatric consultant at a Federal Neuropsychiatric Hospital or university department	A CPN or CHEW trained in mhGAP-IG is posted to one health centre per local government area, receives clinical supervision from a psychiatrist from Federal Medical Centre Makurdi or CBM, and receives additional supervision from the CCMHP Community Mental Health Project Officer
2. Medicine supply Relevant systems for procurement, distribution, storage, quality management and monitoring must be in place to ensure that health centres have an adequate supply of essential medicines	CCMHP procures medicines from CHAN Medi-Pharm and sets up Drug Revolving Fund at each health centre to ensure constant supply. CCMHP Community Mental Health Project Officer is responsible for oversight
3. Outreach Staff should have access to transport to visit clients at home who have complex mental disorders	CCMHP provides each CPN with a motorbike for outreach. Local government is responsible for fuel
4. Training Ensure medical student and nurse training includes common mental disorders, psychosocial interviewing skills and orientation to primary health care. Their curricula of training should be adjusted adequately to include these	CPNs receive formal training and accreditation, funded by CCMHP. Both CPNs and CHEWs receive CCMHP-funded mhGA-IG training and retraining
5. Referrals Clear procedures are required for upward and downward referral, from psychiatric nurses to doctors to specialists, and vice versa	Referrals are made directly between the CPN or CHEW and specialists at Federal Medical Centre Makurdi or Benue State University Teaching Hospital
6. Rehabilitation Social rehabilitation should be encouraged by promoting inclusion in community activities, which might be supported by self-help or peer groups	CCMHP-funded self-help groups and vocational training provide opportunities to combat exclusion and support livelihoods of users and carers. Two community-based rehabilitation facilities also operate under CCMHP
7. Community Engagement with community aspects of primary care system facilitates health promotion, surveillance, referral and follow-up	CCMHP trains lay people as community-level mental health advocates for promotion, identification and referral. CPNs and CHEWs conduct community outreach for follow-up

## Results

### Local context

#### Mental health in Benue State

Located in North Central Nigeria, Benue State is home to more than 5.7 million. Although it is among the top ten most populous states in the country, Benue State is not among the richest. 49% of the population falls into the two lowest quintiles of Nigeria’s wealth index. The majority of the population are engaged in agriculture and live in rural areas [31, 32].

In addition to poverty, a number of other factors may adversely impact mental health in Benue State. For example: sporadic episodes of communal violence and flooding have displaced populations in some areas [33]; thousands of refugees from neighbouring Cameroon have crossed the border into Benue State [34]; and the prevalence of HIV is consistently among the highest in Nigeria [35].

Unfortunately, there is no data available on the prevalence of mental disorders locally. The Nigerian Survey of Mental Health and Well-Being estimates a lifetime prevalence of 12.1% and a 12-month treatment gap of 90% [36]. However, the survey was carried out in predominantly Yoruba-speaking parts of the country, while the majority in Benue State are Tiv, Idoma or Igede speakers. One study of a semi-urban area of Benue State found low rates of epilepsy (4.7 per 1000 population); rates in rural areas are expected to be significantly higher [37].

#### Public and private services

Benue State has two tertiary care facilities, 117 secondary care facilities and 1289 primary care facilities—or approximately 26 health facilities for every 100,000 population. Facilities are not evenly distributed, with most located in urban and semi-urban areas. More than a third belong to the private sector [38]. At the time of CCMHP’s initial situation analysis in 2012, there were already 85 mental health beds in the state, though more than half (49) were located in private facilities. The remainder were in the Psychiatry Department of the Federal Medical Centre staffed by four specialists: three psychiatrists and one clinical psychologist.

The Methodist Church Diocese of Otukpo, Benue State remains the largest private provider of mental health care. The Nigeria Health Care Project, set up in 1992 by the Wesley Guild, established Edawu, a 16-bed community-based rehabilitation centre for homeless people with mental disorders, in 1996 [39]. A 24-bed centre, Agboke-Oglewu, was opened in 2004 with support from the Bishop of the Methodist Church Diocese of Otukpo [40]. Based on the Amaudo model [41], each centre is staffed by a community psychiatric nurse (CPN) and a community health extension worker (CHEW), as well as a community resettlement officer and a workshop manager, and offers inpatient and outpatient care, including community outreach clinics. A predominantly Tiv Christian Reformed Church, NKST (*Nongu u Kristu u I Ser u sha Tar*), also operates a mobile dispensary for psychotropic medications in Mkar, as well as other services in Tiv-majority areas of the state.

### Development of the partnership

Recognising that several of the building blocks for implementation of Nigeria’s mental health policy were already in place in Benue State, CBM International secured five years of funding from the Australian Government’s Department for Foreign Affairs and Trade (DFAT) to launch a PPP in 2011, with an extension into 2016. The aim was to draw together and build upon existing services in the public and non-state sectors to develop a comprehensive mental health system with state-wide coverage, and to reinforce the government’s capacity to operate this system over the long-term. The partnership included state and local government (Benue State Ministry of Health and Human Services, Local Government Service Commission), tertiary care providers (Benue State Teaching Hospital and Federal Medical Centre, Makurdi) and local faith-based organisations (Methodist Church Diocese of Otukpo).

CCMHP’s offices were established on the compound of the Methodist Church Diocese of Otukpo, and are staffed by a ten-person team, including three programmatic staff (Project Coordinator, Self-Help Group Development Project Officer, Community Mental Health Project Officer), five office staff (one Administrator/Finance Officer, a cleaner and three security guards), and two drivers. The programmatic staff spend much of their time in clinics and communities throughout Benue State or at the state capital in Makurdi, working with key stakeholders. A Project Management Committee, with members drawn from among the partners and other key stakeholders, provides oversight.

### Scaling up services

#### Integration into primary care

Nigeria’s Mental Health Policy offers a blueprint for service delivery at each level of the health system, with a special focus on primary care (Table 2). As there were no public mental health services available in primary care at the time that CCMHP was founded, the main focus in this phase was on leveraging partnerships to build capacity, infrastructure and supply chains at this level.

**Fig. 1:**
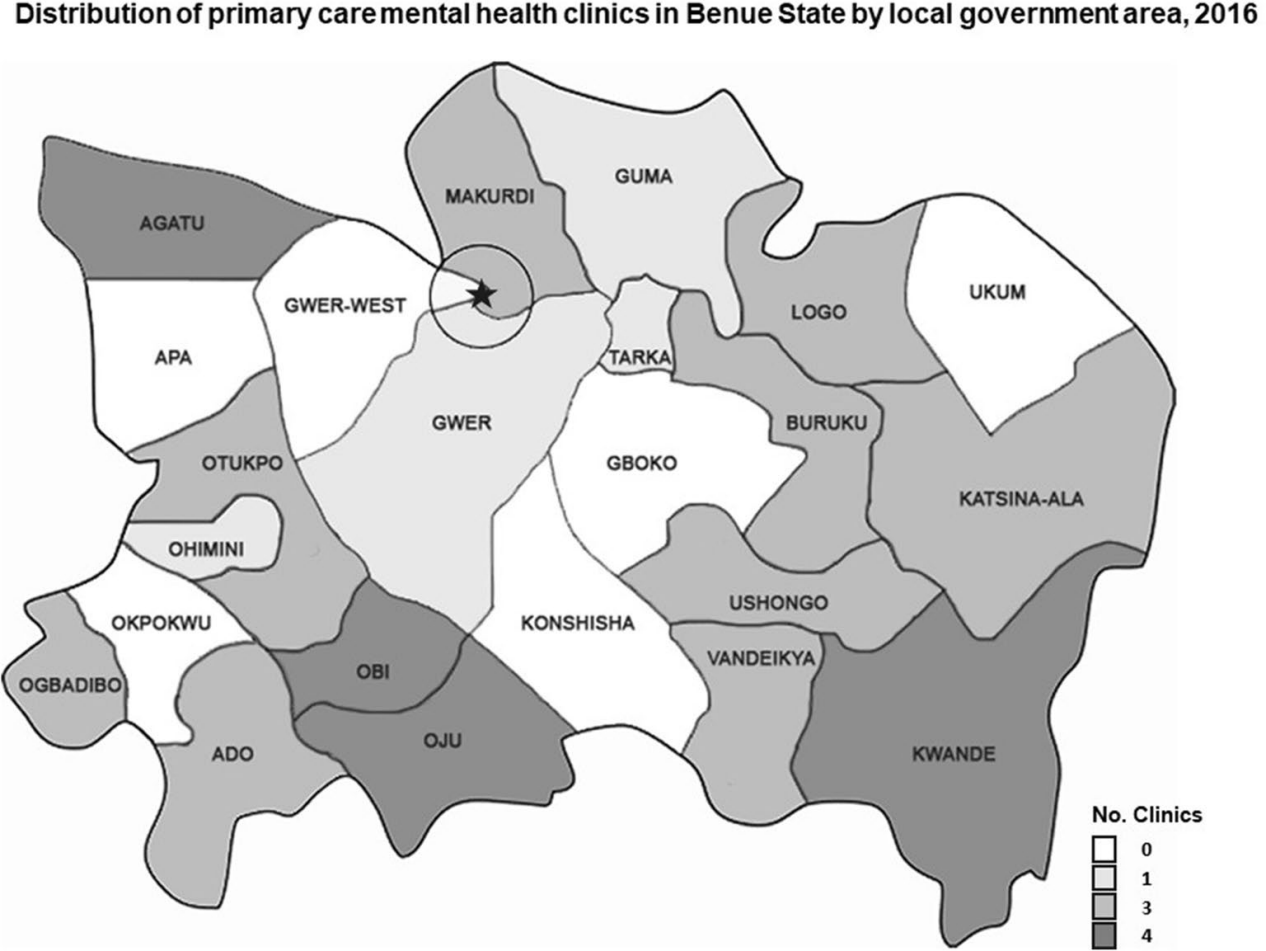
Distribution of mental health clinics in Benue State by local government area, 2016

Local Government Health Authorities were asked to identify nurses at the primary care-level who would be suited to mental health work. CCMHP screened those referred and selected 20 for sponsorships to study community psychiatric nursing at the School of Post-Basic Psychiatric Nursing, Federal Neuropsychiatric Hospital, Kaduna. Nineteen were accredited and further trained in key modules of the WHO’s mental health Gap Action Programme Intervention Guide (mhGAP-IG), alongside 48 community health extension workers (CHEWs) and the two CPNs already operating at Agboke and Edawu. These CPNs and CHEWs established 45 new clinics in primary health centres, in addition to the two private clinics already in Agboke and Edawu. As a result, 17 of the 23 local government areas now have mental health care available from at least one clinic (Fig. 1). (In the next phase, CCMHP intends to have every local government area covered.) CHEWs receive biannual refresher trainings, while CPNs receive annual refresher trainings, sponsored by CCMHP.

CPNs and CHEWs offer on-site clinic days as well as off-site community outreach, whereby clients who have missed their follow-up appointments and those who live far from the clinic are traced, assessed and treated, as needed. CPNs are provided with motorcycles for transport and are entitled to a fuel allowance from the local government, though this is rarely made available in practice. Some also use outreach as an opportunity for awareness-raising and advocacy in local communities, often in collaboration with CCMHP’s trained community-based mental health advocates.

To ensure access to essential psychotropic medicines, CCMHP set up a Drug Revolving Fund, whereby a “seed stock” of medicines is allocated to each mental health clinic. As counterfeit and sub-standard medicines are a significant issue in Nigeria [42], CCMHP generally sources from CHAN Medi-Pharm, run by the Christian Health Association of Nigeria (CHAN). However, as CHAN does not consistently stock all of the medicines required by CCMHP, the programme occasionally purchases from alternative suppliers. Regardless of the supplier, CCMHP sets prices for these medicines at an affordable rate, and the CPN or CHEW is expected to pay all proceeds from dispensing medications back into the Drug Revolving Fund’s bank account, enabling further requisitions. Most clients are able to pay for their monthly supply, at a cost ranging from less than 0.50 to 3.00 USD equivalent monthly, though exceptions are sometimes made for clients in extreme poverty.

In order to maintain quality of care, a psychiatrist from either the Federal Medical Centre in Makurdi or CBM visits each clinic biannually. Quarterly clinical supervision visits were initially planned, with the expectation that a senior psychiatric nurse seconded to CCMHP by the Ministry of Health and Human Services would conduct the additional visits. However, the position was not filled during this phase of the programme. In absence of more frequent clinical supervision, CPNs and CHEWs phone the psychiatrists for additional advice on an as-needed basis, and the Community Mental Health Project Officer visits regularly to supervise non-clinical work, such as record-keeping and supplies management. A Structured Monitoring, Evaluation and Quality Assurance Checklist is completed at each visit by the Community Mental Health Project Officer.

#### Psychosocial support

Self-help groups are common among mental health programmes in sub-Saharan Africa as a means of promoting agency, peer-support, and economic empowerment [28, 43]. During its first phase, CCMHP established 15 self-help groups for service users and carers in 14 different local government areas. One group was severely disrupted by communal violence in the area. Another, initiated in the last few months of phase one, failed to firmly establish itself during this period. The rest continue to operate.

Self-help groups elect their own leadership, with support from the Self-Help Group Development Project Officer. The local CPN or trained CHEW may also be involved in self-help group activities, on an as-needed basis. With funding from CCMHP, self-help groups meet once per month and report on their activities. Self-help groups work to mobilise community resources to address issues of social exclusion. Nine have further developed a rotating loan fund for members, who may borrow approximately 14–140 USD equivalent at a time. CCMHP provides the initial capital investment, along with training in leadership, managing group dynamics, managing finances, business skills and record-keeping, and helps the groups establish clear terms and conditions for their loans. Typically, interest rates range between five and 10 percent, with repayment periods from three to 12 months. Over a three-year period, more than 120 members accessed loans, mostly for farming and petty trading.

In addition to self-help group approaches to livelihoods support, CCMHP offers vocational training for motivated individuals to learn marketable skills. 21 beneficiaries (15 females, 6 males; 20 service users, 1 carer) have attended training centres under this scheme, which covers fees as well as a monthly stipend, as needed. The majority (14) have trained in tailoring, while others have trained in motorcycle repair (2), carpentry (1), poultry farming (1), barbering (1), computer appreciation (1) or health, as a clinic auxiliary (1). CCMHP works with the trainers to ensure reasonable accommodation for service users, outlined in memoranda of understanding (MOUs) between CCMHP, the trainers and the trainees. While CCMHP typically supports one year of vocational training, many skills take longer to master, and beneficiaries are not guaranteed job placements upon completion. During its first phase, CCMHP gave 10 seed grants to help beneficiaries start their own businesses, with plans to do the same for the remainder in its second phase.

#### Advocacy and awareness-raising

The programme launched a State Stakeholder Alliance in 2013, which is comprised of 18 member organisations with an interest in mental health advocacy, including: government ministries, departments and agencies; health institutions; media; faith-based organisations; civil society organisations; traditional governance structures; and users of mental health services. In order to build the capacity of the alliance, five members completed the Mental Health Leadership and Advocacy Programme supported by CBM at the University of Ibadan, Nigeria, between 2012 and 2016 [44]. Membership in the alliance is free, and most of its activities are covered by CCMHP’s budget.

The alliance organises annual celebrations of World Mental Health Day in Benue State, participates in national mental health stakeholder events, engages with media, and regularly visits officials in state and local government as well as health institutions, the Nigeria Prison Service, and others. These activities have proved vital in securing commitments from officials at different levels of government to scale-up mental health services. For example, in 2015 advocacy efforts led to the opening of a new inpatient psychiatric ward at Benue State University Teaching Hospital, after numerous delays handing the ward over to the Department of Psychiatry (due to unpaid debts by the hospital authority to the building contractors). In 2016, the Psychiatry Department of the Federal Medical Centre, Makurdi also agreed to formalise its supervisory role in CCMHP.

In addition to national and state-level advocacy, CCMHP supports community-level awareness raising, which has been shown to increase uptake of mental health services in other parts of Nigeria [45, 46]. Mental health advocates are selected by the CPN and Primary Health Care Coordinator of the local government area, on the basis of the individual’s interest in mental health, residence in the local government area, willingness to undertake advocacy work on a volunteer basis, and ability to read and write in at least one local language. During CCMHP’s first phase, 72 mental health advocates were recruited across 16 local government areas and the catchment areas of two local NGOs.

Using training manuals adapted from Amaudo’s Mental Health Awareness Programme in South-East Nigeria [46], a training of trainers was held for 21 trainers from local government, Benue State University Teaching Hospital, two local NGOs, and CPNs providing services through CCMHP. Mental health advocates were then trained by these trainers over a four-day period. The training covers mental health promotion, warning signs, caring for someone with a mental health condition, referring someone for treatment, and protecting the rights of someone with a mental health condition. Mental health advocates engage with individuals and families as well as with community groups, for example at places of worship, and often distribute printed educational materials. A quarterly stipend equivalent to approximately 8 USD is paid to mental health advocates on the basis of their performance, assessed through quarterly reports verified by the CPN.

By the end of CCMHP’s first phase, 65 mental health advocates remained actively engaged with the programme. According to the programme’s records, mental health advocates reached 38,507 people, mostly females (64.25%), and referred 134, mostly males (53.73%) to mental health services. Anecdotal evidence suggests the efforts of mental health advocates have also led to the release of some individuals who were previously being chained, an abusive practice that is common in West Africa [47].

### Monitoring and evaluation (M&E)

Although Nigeria’s Mental Health Policy calls for “an efficient process of auditing of service provision and delivery for mental, neurological and substance use disorders” [5], mental health has not yet been integrated into the national health management information system, and many mental health programmes are unable to report on basic indicators such as service utilisation [4, 27, 48].

In 2012, CCMHP partnered with researchers at LSHTM to create a paper-based Mental health Information and M&E (MIND ME) system capable of meeting the data needs of diverse stakeholders [29, 30]. Formative research was conducted in 2013 to adapt an existing M&E system which had been developed as part of the Case Studies Project and piloted at two CBM-affiliated sites in southeast and North Central Nigeria. The M&E system is used to collect essential client-level information for service delivery, supervision and—in future—research. This was integrated with a new mental health information system (MHIS) that mimics the national health management information system used for other priority health conditions. The mental health information system generates data on service utilisation disaggregated by sex, age and diagnosis, for monthly reporting to the offices of the Ministry of Health and Human Services and CCMHP, and annual reporting to funders.

The resulting MIND ME system has been in place since 2014 and was revised in 2015. It has been used as a model for other research projects in Nigeria (Emerald, mhSUN) [49, 50], Ghana (BasicNeeds) and Uganda (Brain Gain II) [51]. A generic version is currently being developed for wider dissemination.

#### MHIS data

MHIS data from the first phase of CCMHP’s activities indicates 13,785 clients were enrolled across 47 mental health clinics between 2011 and 2016, an average of 282 per year per clinic. Most were male (54.93%) and over the age of 18 (82.75%) (Table 3).

**Table 3:** Demographic characteristics at enrolment, 2011–2016

Demo-graphics	2011^a^		2012^a^		2013^a^		2014		2015		2016		Subtotals	
	n	%	n	%	n	%	n	%	n	%	n	%	n	%
Female	948	46.54	1143	42.84	981	43.79	657	46.79	1315	46.90	1169	44.41	6213	45.07
Child	134	6.58	22	0.82	42	1.88	175	12.46	377	13.45	327	12.42	1077	7.81
0–5	–		–		–		14		38		45			
6–12	–		–		–		54		129		120			
13–17	–		–		–		107		210		162			
Adult	814	39.96	1121	42.02	939	41.92	482	34.33	938	33.45	842	31.99	5136	37.26
18–25	–		–		–		183		388		336			
26–35	–		–		–		191		303		249			
36–50	–		–		–		77		191		185			
51+	–		–		–		31		56		72			
Male	1089	53.46	1525	57.16	1259	56.21	747	53.21	1489	53.10	1463	55.59	7572	54.93
Child	174	8.54	49	1.84	64	2.86	198	14.10	418	14.91	398	15.12	1301	9.43
0–5	–		–		–		18		62		72			
6–12	–		–		–		82		149		144			
13–17	–		–		–		98		207		182			
Adult	915	44.92	1476	55.32	1195	53.35	549	39.19	1071	38.20	1065	40.46	6271	45.49
18–25	–		–		–		194		414		399			
26–35	–		–		–		222		396		390			
36–50	–		–		–		88		173		198			
51+	–		–		–		45		88		78			

^a^2011–2013: No MIND ME system in place

Data disaggregated by age group and diagnosis is only available after 2014, when the MIND ME system was put in place. Substantial quantities of data are missing from the year 2014 while the transition to MIND ME was underway. However, data available from 2015 to 2016 suggests that most clients fall within the 18–25 (24.32%) or 26–35 (24.61%) age groups, which is also reflected in the low proportion of child mental disorders (0.29%) and dementia (0.16%) recorded. Epilepsy (52.38%) and psychosis (38.41%), a category which includes bipolar disorder, are predominant, though there does appear to be a very slight increase in the percentage of clients enrolling for “common mental disorders” (depression or anxiety) year on year (Table 4).

**Table 4:** Clinical characteristics at enrolment, 2014–2016

Diagnosis	2014		2015		2016		Subtotals	
	n	%	n	%	n	%	N	%
Epilepsy	687	48.41	1518	53.75	1396	53.04	3601	52.38
Psychosis/bipolar	593	41.79	1057	37.43	991	37.65	2641	38.41
Depression/anxiety	81	5.71	173	6.13	183	6.95	437	6.36
Alcohol/substances	39	2.75	50	1.77	51	1.94	140	2.04
Developmental	10	0.70	12	0.42	3	0.11	25	0.36
Child mental disorder	7	0.49	9	0.32	4	0.15	20	0.29
Dementia	2	0.14	5	0.18	4	0.15	11	0.16

Rates of referral in and out of CCMHPs’ clinics are low (Table 5). Observation of clinics’ referral registers and follow-up queries with providers suggest the low rate of referrals to clinics from other services (0.39%) and from the clinics to other services (0.32%) is not due to missing data, but rather reflects on-the-ground realities of referral patterns.

**Table 5:** Referrals in and out of clinics, 2014–2016

Type	2014		2015		2016		Sub-total	
	N	%	n	%	n	%	N	%
Referral in	8	0.57	30	1.07	16	0.61	54	0.39
Referral out	11	0.78	23	0.82	10	0.38	44	0.32

### SWOT analysis

#### Strengths

Since 2011, CCMHP has made significant strides toward the implementation of Nigeria’s Mental Health Policy. It has created links between communities and services, between public and private services, between primary and tertiary services, and between key stakeholders state-wide. It has rapidly scaled up non-specialist mental health services by establishing new clinics staffed by CPNs and CHEWs trained in mhGAP-IG. It has also engaged specialists at Benue State University Teaching Hospital and the Federal Medical Centre in Makurdi in clinical supervision, advocated with other stakeholders for the opening of a new psychiatric unit at Benue State University Teaching Hospital, and forged new relationships with social services, such as the Ministry of Education and the Nigeria Prison Services. Perhaps most importantly, it has maintained these efforts over periods of political instability—including elections, defaults on public sector salaries, widespread industrial action, and periodic episodes of communal violence—which might otherwise have crippled the programme in the absence of strong partnerships with non-state actors.

Nevertheless, there remains room for improvement, as is evident from the patterns of service utilisation reported by CCMHP clinics. Additionally, the inability of state and local governments to fulfil some of their obligations to the programme threaten its future sustainability, as described further below.

#### Weaknesses

The high proportion of epilepsy and psychosis among CCMHP’s clients is consistent with observations from other community mental health programmes in sub-Saharan Africa [52], including a CBM-supported programme in Abuja [27]. Although there was no formal referral pathway in place for complex cases in Abuja, Benue State has engaged both Benue State University Teaching Hospital and Federal Medical Centre, Makurdi. The low rates of referral reported via the MHIS, combined with high rates of so-called “severe mental disorders” like psychotic disorders, could indicate a tendency among CPNs and CHEWs to go beyond their remit as non-specialists. In interviews, some CPNs and CHEWs suggested the barriers to accessing tertiary care in Makurdi are so great, they feel obligated to treat even complex cases, rather than referring them to specialist care.

Meanwhile, in the absence of a full-time, senior psychiatric nurse seconded to the programme, CCMHP has not met its target of conducting a quarterly clinical supervision visit to every clinic. While the Mental Health Policy proposes that primary care physicians fill the gap between CPNs, CHEWs and specialists in tertiary care—both in terms of clinical supervision and referral pathways—in practice, there are exceptionally few physicians working in primary care facilities [4], and secondary care facilities were not engaged in CCMHP’s first phase. These are issues which must be addressed in order to ensure quality of care going forward.

#### Opportunities

Although it is estimated that approximately half of all lifetime mental disorders start by the mid-teens [53], the relatively low proportion of clients under 18 who were enrolled in phase one indicates that CCMHP should focus especially on identifying opportunities to strengthen child and adolescent mental health in future. School-based approaches would be in-line with Nigeria’s Mental Health Policy, which advocates for more partnership between the health and education sectors to enable mental health promotion, early detection, treatment and rehabilitation, and suicide prevention for young people in schools and universities [5]. It is worth noting that child and adolescent mental health is an area of special interest for many funders. For example, the Wellcome Trust’s new 200 GBP million priority area on mental health research focuses on child and adolescent mental health, particularly depression and anxiety [54]. There may be opportunities for CCMHP to identify new funding to support further work in this area.

#### Threats

DFAT has agreed to fund a second phase of operations for CCMHP, offering an important opportunity to build on new relationships and address issues encountered in phase one. However, there is also an expectation that the programme will become self-sustaining in the next five years by transitioning entirely to public sector funding and management. Yet recent estimates from WHO indicate only 27% of countries in the African region have actually allocated the human and financial resources outlined in their mental health policy or plan [55]. Given that state and local government in Benue have defaulted on some commitments outlined in the original MOUs, government’s ability to deliver over the long-term is called into question, as is CCMHP’s ability to continue operations once external funding is withdrawn.

For example, local governments signed MOUs with CCMHP in which they agreed to provide essential equipment at start-up, such as secure storage for medical records and medications, plus a monthly allowance to cover clinics’ ongoing operational costs, such as printing MIND ME forms and fuel for outreach. These commitments have not been consistently honoured in practice.

At the state level, the Ministry of Health and Human Services has been able to integrate mental health into its existing portfolio of work but has been unable to allocate new resources to mental health. For example, mental health was added to the existing portfolio of work of a Sexual and Reproductive Health Officer, in order to create the role of Mental Health Desk Officer within the Department of Public Health. However, during phase one the Ministry did not second a senior psychiatric nurse to serve as the Clinical Officer, a crucial supervisory role. Similarly, nurses already in-post in primary care who were then trained as CPNs were able to start offering mental health services, but the additional nurses trained by CCMHP were not given placements by the Ministry.

In several cases, CCMHP has overcome institutional inertia by meeting costs that were not covered by local and state government, but this is not a sustainable solution in the long-run. Further, it risks establishing a precedent whereby government is absolved of responsibility for delivering on its commitments. Nigeria’s Mental Health Policy specifies that funding for primary, secondary and tertiary care is the responsibility of the local, state and federal government, respectively; however, this may not be realistic without substantial improvements in governance and accountability in the public sector, and particularly at the level of local government. CCMHP would not be the only mental health programme initiated as a PPP to falter after being handed over to government in a low-resource, conflict-affected setting [52]. CCMHP’s non-state partners lend continuity in a challenging context, in which political instability is a constant threat.

## Discussion

This case study describes a Nigerian PPP that has achieved a rare but much-desired outcome in global mental health [56]: the rapid scale-up of mental health in primary care, in line with national mental health policy and the WHO’s mhGAP. Much of the literature on PPPs in global health focuses on international partnerships with for-profit entities [15, 23–25]. CCMHP offers a promising example of partnership between local and state government and faith-based organisations, with funding and coordination managed by an international NGO. This structure has several advantages.

First, faith-based organisations play an important role in healthcare in sub-Saharan Africa. For example, a meta-analysis by Kagawa, Anglemyer and Montagu (2012) estimates that in sub-Saharan Africa, 6.8% of all deliveries take place at facilities run by faith-based organisations [57]. A review by Widmer and colleagues (2011) of maternal and newborn health services in Africa concludes that those provided in the public sector are similar to those provided by faith-based organisations, but faith-based organisations may provide better quality of care and result in higher levels of satisfaction with services [58]. The authors also comment that facilities operated by faith-based organisations often remain active even during times of political instability and humanitarian crisis [58]. While there is very little research published on the delivery of mental health care by faith-based organisations in LMICs, it is worth noting that the Methodist Church’s two community-based rehabilitation centres have each been operating continuously for at least 15 years in Benue, with high demand for their services.

Second, NGOs play a particularly important role in channelling resources to mental health in LMICs. Private philanthropy to NGOs and foundations is the single largest source of overseas development assistance for health directed to mental health: 435 USD million in the years 2000–2015 [59]. Combined with other funding—for example, from bilateral aid agencies—NGOs and foundations channel approximately two-thirds of all the overseas development assistance for health spent on mental health in LMICs [59]. Large international NGOs like CBM may be seen as “safe hands” to manage finances in countries perceived by funders as having high levels of government corruption [60]. While human rights watchdogs have praised recent strides in combatting public sector corruption [61], Nigeria topped Transparency International’s list of the world’s most corrupt countries at the start of the new millennium, and it remains in the top 20% of countries on the Corruption Perceptions Index [60, 62].

Third, PPPs with not-for-profits may also side-step growing concerns regarding the engagement of for-profit entities with the potential to distort power relationships in health partnerships [24]. Indeed, Iemmi’s (2019) recent mapping of external actors in global mental health calls for more multisectoral collaboration, while simultaneously warning of “new ethical challenges spurred by financial motives” (pp. 7) [63]. However, potential conflicts of interest are also possible in partnerships with non-secular and other not-for-profit partners. It is notable, for example, that the Tiv Christian Reformed Church (NKST) has chosen to keep an arm’s length from CCMHP, which may perhaps be perceived as a mainly Methodist partnership.

The most obvious drawback to the CCMHP partnership is related to sustainability. Nigeria has identified PPPs as desirable mechanisms “for providing, and funding, services” (pp. 22) [5]. Indeed, the need for creative solutions to compensate for shortfalls in public spending is what catalysed the rapid uptake of PPPs in high-income countries in the late twentieth and early twenty-first centuries, and one of the main drivers in LMICs today [19]. While CCMHP demonstrates that a PPP with not-for-profits can support the provision of mental health services at scale, its funding is largely dependent on time-limited development assistance. This represents a significant risk, particularly in light of repeated cuts to Australia’s foreign aid budget in recent years [64]. More research is needed to investigate sustainable alternatives, which may perhaps include involvement of for-profit partners.

## Conclusion

The case of CCMHP illustrates that it is in fact possible to leverage a PPP with not-for-profit partners to rapidly expand mental health services in primary care, as part of broader efforts to implement mental health policy. However, coordinated action, based on realised commitments, is needed across the primary, secondary and tertiary levels of healthcare. Further, there is a need to take into consideration challenging political contexts when planning to transition a PPP to full public sector ownership. It may be that neither the public nor the not-for-profit private sector—nor even a combination of the two—is ready to sustainably finance service delivery at scale in these settings over the long-term. More research is needed to document and evaluate PPPs for mental health in LMICs, with a focus on sustainability [27]. Lessons learned would be relevant not only to the scale-up of mental health services in Nigeria, but also to other LMICs that are working to transform mental health policy into reality.

## Data Availability

The MHIS data that support the findings of this case study are available from CCMHP, but restrictions apply to these data, which were used under MOU for the current study. MHIS data are however available from the authors upon reasonable request and with permission of CCMHP.

## Abbreviations

CCMHP: Comprehensive Community Mental Health Programme
CHAN: Christian Health Association of Nigeria
CHEW: Community health extension worker
CPN: Community psychiatric nurse
DFAT: Department for Foreign Affairs and Trade
GBP: British sterling
LMICs: Low- and middle-income countries
LSHTM: London School of Hygiene and Tropical Medicine
M&E: Monitoring and evaluation
mhGAP-IG: WHO mental health Gap Action Programme Intervention Guide
MHIS: Mental health information system
MIND ME: Mental Health INformation anD Monitoring and Evaluation
MNS: Mental, neurological and substance use
MOU: Memorandum of understanding
NKST: *Nongu u Kristu u i Ser u sha Tar* (Tiv Christian Reformed Church)
PPP: Public–private partnership
USD: United States Dollars
WHO: World Health Organisation
